# Subretinal Aspects of the Optoretinographic Response

**DOI:** 10.1167/iovs.67.6.21

**Published:** 2026-06-12

**Authors:** Reddikumar Maddipatla, Maciej M. Bartuzel, Ewelina A. Pijewska, Christopher S. Langlo, Robert J. Zawadzki, Ravi S. Jonnal

**Affiliations:** 1 Center for Human Ophthalmic Imaging Research (CHOIR), UC Davis Eye Center, Sacramento, California, United States; 2 EyePod Small Animal Ocular Imaging Laboratory, Department of Cell Biology and Human Anatomy, UC Davis, Davis, California, United States; 3 Department of Biomedical Engineering, Wroclaw University of Science and Technology, Wybrzeze Wyspianskiego, Wroclaw, Poland

**Keywords:** optoretinography, ORG, OCT, subretinal space, supracone space, retinal imaging, photoreceptor function

## Abstract

**Purpose:**

Optoretinography (ORG) detects stimulus-evoked, nanometer-scale changes in the optical path length of photoreceptors, hypothesized to reflect osmotic water shifts into and out of the outer segments (OS), as well as electrostatic effects of opsin photoisomerization. The aim of this study was to measure parallel changes in the subretinal space (SRS), which may reflect changes in the SRS volume. The results of these experiments could affect interpretations of the photoreceptor ORG, which make testable hypotheses about the SRS volume. Moreover, because water movement in the outer retina is impeded in a number of diseases of the outer retina, this method could represent a novel biomarker of outer retinal health.

**Methods:**

A custom swept-source optical coherence tomography system (100 kHz, 1060 nm) was used to image the eyes of four healthy subjects. After dilation and dark adaptation, serial B-scans were acquired over 125 ms at 400 Hz, with a stimulus flash delivered after 40 ms. From the resulting series, relative phase velocities between layers were calculated for three compartments: OS, supracone space (SCS), and ciliary zone (CZ).

**Results:**

Light stimulation produced rapid, layer-specific responses. Following stimulation, the OS contracted briefly and then elongated, the SCS elongated and subsequently contracted, and the CZ exhibited a response similar to the OS but with lower amplitude. These responses were temporally coordinated across layers, suggesting coupled structural and fluid dynamics within the outer retina.

**Conclusions:**

Visible light induces rapid, reversible structural changes across multiple outer retinal layers, including the spaces surrounding the photoreceptor outer segments. Noninvasive measurement of these responses may improve understanding of the biophysical sources of the ORG signal and provide a probe of outer retinal functional integrity.

In ophthalmic clinics, diseases affecting the outer retina, such as AMD and geographic atrophy (GA), RP, and ABCA4-associated cone–rod dystrophy, are assessed by observing structural abnormalities in the retinal layers using optical coherence tomography (OCT). However, assessment of retinal function may provide more timely information about the progression of these diseases, improving clinical management and streamlining drug discovery. Traditionally, photoreceptor function is assessed using visual acuity, visual field tests, and electroretinography (ERG). These methods are indispensable but they do not provide simultaneous structural and functional information. Optoretinography (ORG) is an emerging class of methods designed to measure stimulus-evoked changes in retina using noninvasive optical imaging. Photoreceptor structure and functional response have been measured together in cones^1^^–^^5^ and rods^6^^,^^7^ using advanced ORG systems with adaptive optics (AO) or digital aberration correction. However, clinical translation of AO-based imaging systems is hampered by their requirements for costly components, large amounts of space, multiple skilled operators, large capacities for data processing and storage, and lengthy measurement durations. Fortunately, novel, proto-clinical ORG approaches using custom OCT systems very similar to those currently used in clinics have been reported recently.^8^^–^^10^

To date, most ORG work in the outer retina has measured light-evoked changes in photoreceptor outer segments. Generally this consists of measuring responses from single cells, most commonly by monitoring the phase difference between cone/rod outer segment (OS) tips (COST or ROST) and the inner–segment OS junction (ISOS). Changes in the phase difference between these structures indicates a change in the optical path length of the OS. This change, which we refer to as ΔOPL_OS_ here, has been the predominant source of signal in the ORG literature. Investigators have tended to interpret ΔOPL_OS_ as a change in the geometric length of the OS without significant refractive index changes. Transverse imaging of dissected OSs during light exposure has provided some evidence that this is the case,^11^^,^^12^ but further work is necessary for a conclusive interpretation. In this work, we use terms derived from geometric length, such as ‘elongation’ and ‘contraction,’ but we remain agnostic about the precise mechanisms and nature of ΔOPL_OS_ and related observations.

In the present work, we used a proto-clinical, velocity-based approach,^8^^,^^13^ to measure ORG responses inside and outside the photoreceptors in parallel, including boundaries of the OS (ISOS and COST) and the RPE. Specifically, we measured changes in thickness of three layers: ISOS–COST, COST-RPE, and ISOS–RPE. As mentioned, the ISOS–COST distance is the length of the cone OS. The two other lengths we studied do not have neat anatomical counterparts. The COST–RPE length includes extracellular matrix (ECM), RPE apical processes (APs), and potentially phagosomes containing shed OS discs, while the ISOS–RPE length includes those components along with the OS. The first of these, the layer bounded by the COST and the RPE, has sometimes been called the supracone space.^14^^,^^15^ Some investigators use the term subretinal space (SRS) to denote the region containing ECM, AP, and phagosomes, whereas others use it interchangeably with ECM. Figure 1 is a diagram illustrating these lengths and their constituents.

**Figure 1.**
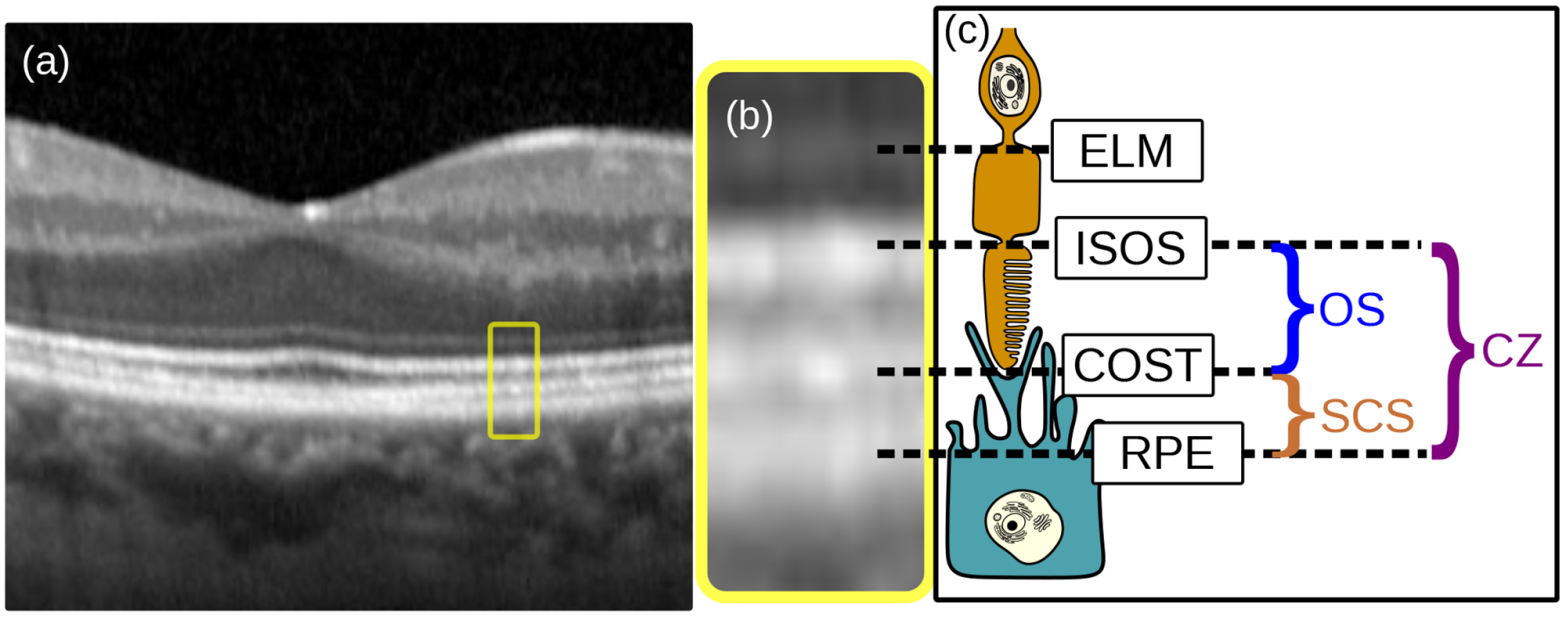
Compartments of the outer retina investigated in this study. (**a**) An OCT B-scan through the foveal center. A *yellow box* highlights a region of the B-scan containing the four outer retinal bands. (**b**) A magnified view of the same region. (**c**) A diagram of the cells believed to contribute to these bands: a cone photoreceptor (*orange*) and a RPE cell (*blue*). The specific cellular structures believed to generate the bands are labeled: the ELM, the ISOS of the cone, the COST, and RPE. The last three of these demarcate the compartments investigated in the present study. The OS is bounded by the ISOS and COST. The SCS is bounded by the COST and the RPE. The CZ is bounded by the ISOS and the RPE.

The use of the conflicting prefixes “sub-” and “supra-” to describe material that is outer to the retina and cone, respectively, is a historical accident. The former originates in 19th-century clinical ophthalmic histology,^16^^,^^17^ and the latter originates in neurobiological histology,^18^^,^^19^ where the outer retina is traditionally positioned at the top of micrographs. In OCT-based anatomical studies, which position the outer retina at the bottom of the image, “subretinal space” is an intuitive name for material lying below the neural retina. Accordingly, “subcone space” may be more intuitive than “supracone space.” Fortunately, both can be abbreviated “SCS,” which is what we use to designate it. To our knowledge, the other length investigated in this study, the layer bounded by ISOS and RPE, has no accepted name. We will refer to it as the ciliary zone (CZ). This is a provisional name, used here only to permit description of the measured quantities and their relationships to the SRS and potential changes in its hydration.

Light-induced movement of water in the outer retina has been investigated in a number of animal models,^20^^–^^25^ but it has not yet been measured in the living human eye. Generally, water enters the retina via aquaporin-4 channels in the vitreous-facing end feet of Muller cells^26^ and is excreted into the ECM of the SRS. From the SRS, fluid is cleared primarily through active transport across the RPE and pulled into the choriocapillaris by osmotic pressure. The full functional significance of this flow is unknown, but it has been shown that it is necessary for the adhesion of the neural retina to the underlying RPE.^27^ Accumulation of fluid in the retina is associated with a number of retinal disorders and diseases, such as retinal detachments, central serous retinopathy, AMD, Stargardt’s disease, and diabetic retinopathy.^27^^–^^29^

In four subjects without retinal disease, we observed the following after stimulus flashes: (1) the OS exhibits a brief (<20 ms) contraction followed by a longer elongation; (2) the SCS length exhibits a brief (<20 ms) elongation followed by a longer contraction, complementary to the OS change but with smaller magnitude; and (3) the CZ exhibits a pattern of change similar to the OS but smaller in magnitude.

Light exposure is known to increase the volume and water content of the ECM in the SRS,^20^^,^^30^ but the methods used to establish that effect in animal models—concentration tracers and osmometry—are not suitable for human studies. Light-evoked increase in ECM hydration is a potentially significant process because it takes place in the context of a complex, highly regulated system of water movement from the SRS through the RPE and into the choroid. That system is disturbed in a number of diseases of the RPE–Bruch’s membrane complex, most notably AMD.^31^ It is very likely that the profound changes seen in Bruch’s membrane permeability in the aging and disease-affected eye would affect the dynamics of light-evoked SRS hydration. Changes in SCS and CZ cannot be unambiguously attributed to changes in the volume of a particular anatomical structure, because both spaces consist of heterogeneous material, both cellular (OS and AP) and acellular (ECM, phagosomes). However, because light-evoked SRS hydration is a well-known phenomenon, we believe it is likely to play a role in light-evoked changes in the SCS and CZ, and this hypothesis is supported by the observations described herein.

## Methods

The OCT system has been described in detail in previous publications.^8^^,^^13^ In short, it consists of a 100-kHz swept-source laser (Axsun Technologies, Billerica, MA, USA; λ = 1060 nm; Δλ = 100 nm), two galvanometric scanners (Cambridge Technologies, Atlanta, GA, USA), a transmissive reference arm, a balanced photoreceiver (Thorlabs, Newton, NJ, USA), and a digitizer (Alazar, Pointe-Claire, Quebec, Canada). A 90/10 fiber coupler sent 90% of the light to the reference arm and 10% to the eye. The back-scattered light from the eye was combined with the reference light using a 50/50 fiber coupler, and a retroreflector was translated in one dimension to adjust the reference arm length. The power of the illumination source, measured at the cornea, was 1.8 mW.^8^^,^^13^ The stimulus channel consisted of a 555-nm LED, selected because that wavelength stimulates the L and M cones approximately equally. The right eyes of four healthy subjects were imaged, aged 29 to 50 years. The research was conducted in compliance with the Declaration of Helsinki and with approval from the University of California Davis Institutional Review Board. Subjects are referred to by anonymized, Institutional Review Board-assigned identification numbers that are unique across multiple ongoing studies in our laboratory and therefore are not consecutive within this study. Before imaging, the angles and IOPs of each subject were measured to ensure a low risk of acute open-angle glaucoma due to dilation. Then eyes were dilated using tropicamide 1%. Subjects were dark-adapted for 5 minutes before imaging. In each subject, five ORG measurements were acquired at each of five retinal eccentricities: 2°, 4°, 6°, 8°, and 10°. Measurements were arranged along iso-eccentric arcs on the temporal retina, distributed by varying polar angles while maintaining a constant radial distance from the foveal center. Each measurement consisted of a series of 50 B-scans acquired at a single location at a rate of 400 Hz, with 16 B-scans (40 ms) acquired before stimulus onset and 34 B-scans (85 ms) acquired after stimulus onset. Each B-scan consisted of 250 A-scans, with a lateral sampling interval of 3 µm and spanned a 2.5° field of view (approximately 750 µm on the retina). A circular stimulus flash with a 1.2° field of view (approximately 360 µm on the retina), covering approximately one-half of the B-scan extent, and centered on it, was delivered. Quantitative analysis was restricted to the first 40 ms following stimulus onset, and most plots shown below were constrained to this range. Beyond 60 ms, phase noise appeared to increase in all trials, potentially due to reflexive eye movements after the stimulus. Therefore, later time points were excluded from both the quantitative analysis and plots herein. Integration of the velocity signals tends to suppress noise, and thus conveys the behavior of the three compartments over longer intervals.

**Figure 2.**
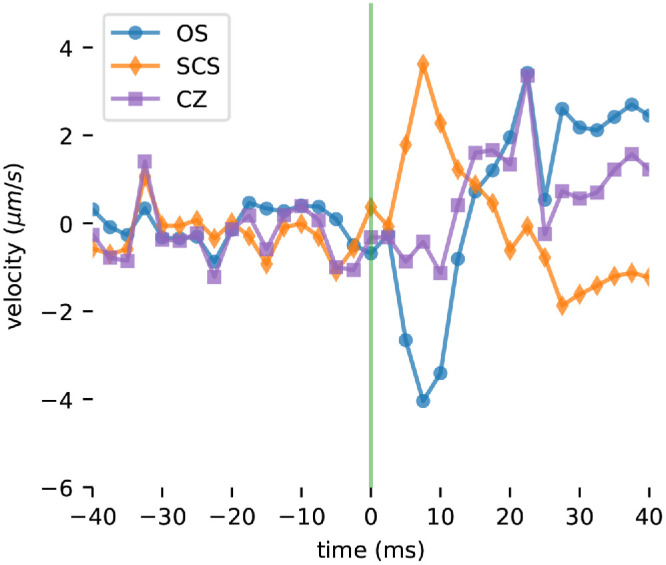
Changes in lengths of the OS (COST–ISOS), SCS (RPE–COST), and CZ (RPE–ISOS) in response to a visible stimulus. The relative velocity between the ISOS and COST is the rate of contraction/elongation of the OS (*blue line*). As previously reported, after stimulus onset, the OS exhibits a rapid contraction followed by a slower period of elongation. The relative velocity of the SCS (*orange line*), between the COST and RPE, exhibits opposing changes: an initial elongation followed by a slower contraction. The CZ (*purple line*) exhibits a change similar to the OS, but with a lower magnitude of contraction. The CZ response is the sum of the OS and SCS responses, a consequence of the phase differences used to calculate relative velocities. Plotted data are the result of a single trial from subject 1 at a location 2° temporal to the fovea. The vertical *green line* indicates stimulus onset.

**Figure 3.**
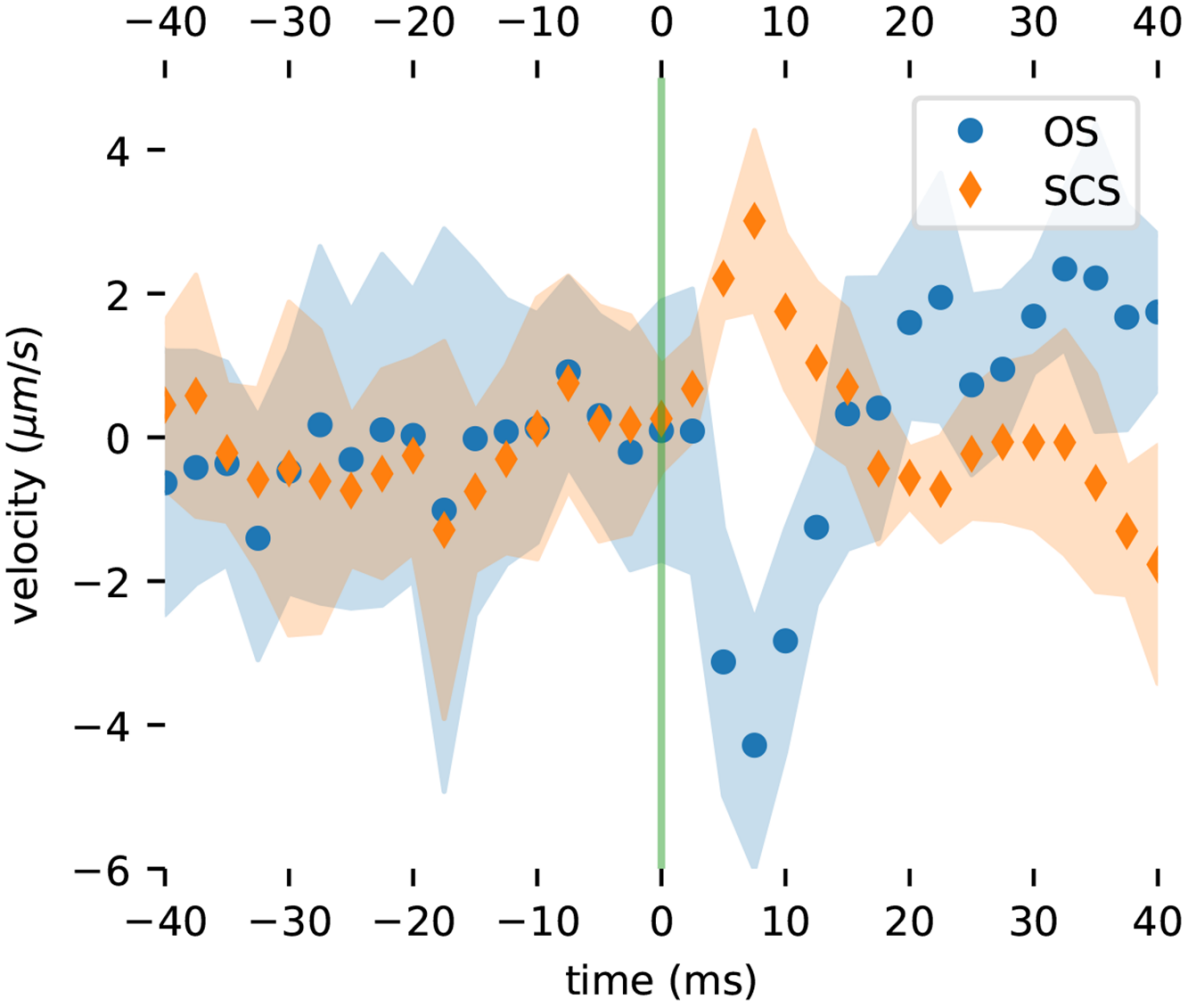
Averaging multiple trials to improve signal-to-noise ratio. Multiple trials were acquired at each eccentricity in each subject to facilitate the quantification of the OS and SCS changes. Averaging these together improves the signal-to-noise ratio and the visibility of the signals and their relationship. Plotted here are the average of five trials at 2T in subject 9. Responses of the OS and SCS are shown (*blue circles* and *orange diamonds*, respectively), along with standard deviation of the trials (*blue and orange shaded regions*, respectively). While the two responses have opposite signs, they do not appear to be fully complementary. The initial SCS expansion is smaller in amplitude than the corresponding OS contraction.

**Figure 4.**
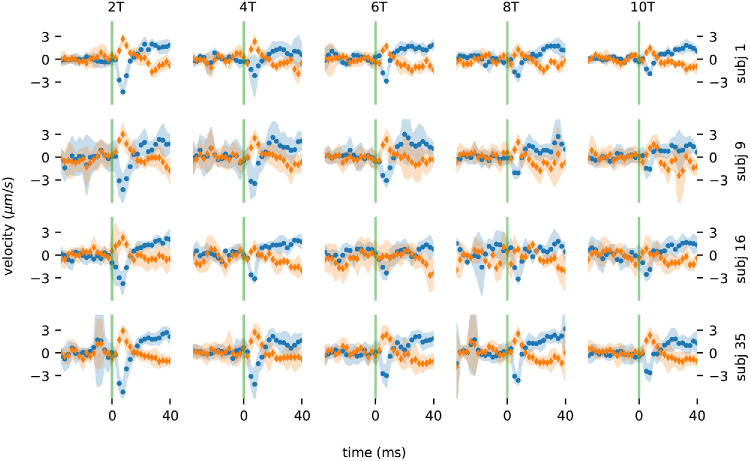
Responses from all subjects at all eccentricities. Plotted here are the rates of OS (*blue*) and SCS (*orange*) length change for all tested subjects and eccentricities. The individual responses and eccentricity dependence of responses were consistent among subjects.

**Figure 5.**
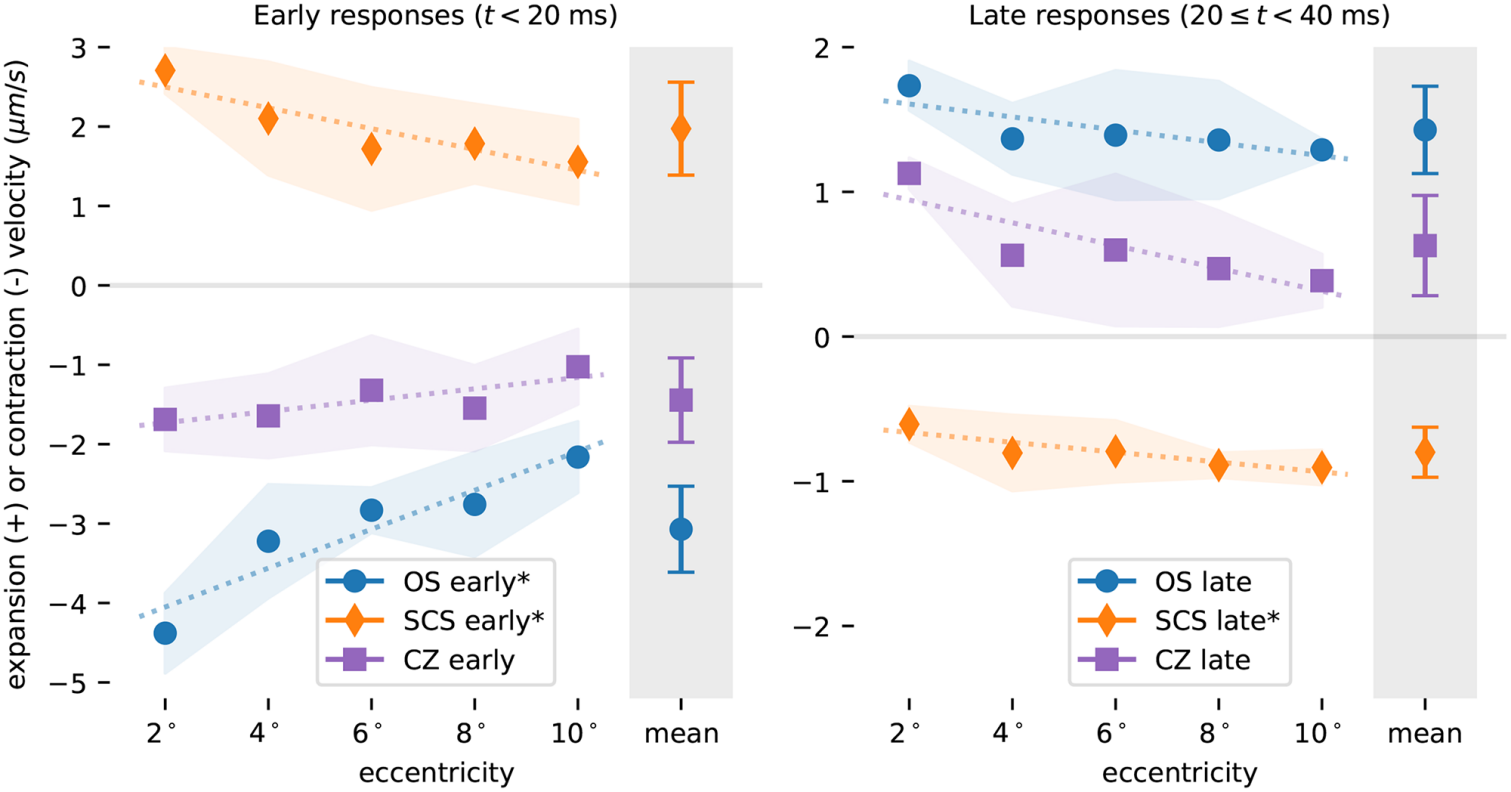
Response parameters as a function of eccentricity. Plotted here are summaries of the responses, averaged across subjects and plotted against eccentricity. Each response was quantified in the early (<20 ms, *left*) and late (20 ms to 40 ms, *right*) stages of response, using extrema or mean values, respectively. Each marker represents the average parameter across subjects, with *filled regions* representing the standard deviation. Three of the parameters (OS early, SCS early, and SCS late) exhibited statistically significant (*P* < 0.05) correlations with retinal eccentricity. All parameters exhibited marginally significant correlations (*P* < 0.1). The three responses, in the early and late windows, should be interpreted as interdependent, due to the commonality of the phase values used to calculate them. The CZ signal is the sum of the OS and SCS signals. *Dashed lines* represent linear fits to the data. Early and late responses were assessed using predefined temporal windows (<20 m and 20–40 ms), rather than discrete time points, to improve robustness to noise and mitigate the effects of limited temporal sampling in individual traces.

**Figure 6.**
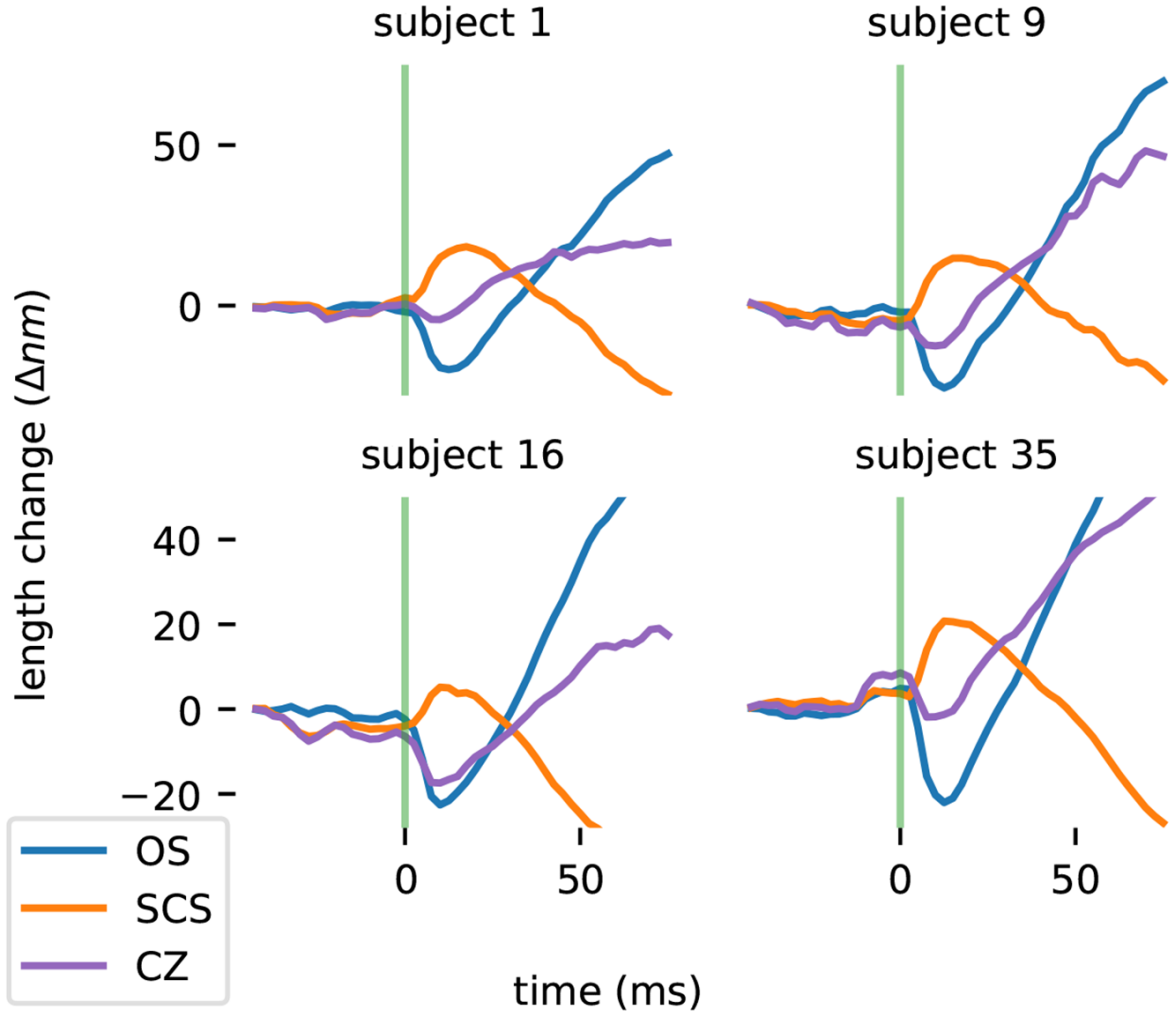
Lack of reciprocity of OS and SCS responses. Shown in these plots are the cumulative sums (numerical integrals) of relative velocities, which should correspond with length changes of the relevant zones. Length changes are plotted for the OS and the SCS, as well as the sum of these two responses, averaged across the 2° and 4° eccentricities, for each of the four subjects. In all subjects, the sum of the two individual zone responses is non zero, indicating that the OS and SCS responses are not reciprocal.

### Stimulus Delivery and Photopigment Bleaching

A circular 1.2° stimulus flash was delivered 40 ms after measurement initiation using a fiber-coupled green light-emitting diode (LED; MINTF4, Thorlabs; nominal peak wavelength 554 nm). The LED was driven by a multi channel LED controller (DC4100, Thorlabs), which provided electronic gating of the stimulus. The controller was operated in external control mode (0–100 kHz modulation bandwidth). Given this bandwidth, the rise and fall times are expected to be on the order of microseconds and therefore negligible relative to the 30-ms stimulus duration. Light exiting the fiber was collimated and spectrally filtered using a 23-nm bandpass filter centered at 555 nm (FF01-554/23; Semrock, Lake Forest, IL, USA), defining the effective stimulation bandwidth. The radiant power at the pupil was 44 µW and the stimulus duration was 30 ms. Photopigment bleaching was estimated using a standard exponential bleaching model,^32^ following previously reported methods.^8^ To improve transparency and reproducibility, the bleaching calculation framework, assumptions and parameter values used in this study are provided in a publicly available photopigment bleaching simulation worksheet (Zenodo repository: [https://doi.org/10.5281/zenodo.18988166]).^32^ Under these conditions, the stimulus was estimated to bleach approximately 65% of L- and M-cone photopigment within the stimulated region, based on the exponential bleaching model described elsewhere in this article.

OCT signal processing was done using standard swept source OCT signal processing methods. Retinal layer velocities were computed using previously reported methods,^8^^,^^13^ by analyzing three consecutive B-scans at a time. B-scans were aligned using amplitude-based cross-correlation and phase-based bulk motion correction. ISOS, COST, and RPE layers were segmented in a semi-automated way, and phase velocities were calculated at each layer in each lateral point in the segmented region. Relative velocities between the layers were calculated next, and then integrated laterally over the stimulated region to obtain the length change. OS and SCS elongation/contraction rates were calculated by subtracting the ISOS velocity from the COST velocity, and the COST velocity from the RPE velocity respectively; the CZ velocity was defined as the sum of the OS and SCS velocities (which is numerically identical to the difference between the RPE and ISOS velocities).

## Results

Consistent patterns of response were found in the OS, SCS, and CZ in all subjects. After stimulus onset, the OS band contracted within the first 15 ms, and then elongated from 20 to 40 ms. The SCS exhibited elongation and contraction, respectively, during these periods. The CZ exhibited changes similar to the OS, but with a smaller magnitude. An example of these characteristic behaviors is shown in Figure 2, which shows the OS, SCS, and CZ dynamics for subject 1 from a single trial at one location 2° temporal to the fovea.

Figure 3 presents averaged responses from five different locations at 2° temporal from subject 9. Here, differences between the OS and SCS responses are visible. The OS contracts while the SCS expands, and the magnitude of the former is greater than that of the latter. In this trial, the peak (contracting) velocity of the OS and the peak (expanding) velocity of the SCS both occurred 8 ms after stimulus onset, but in some trials the former preceded the latter by up to 2 ms. The magnitude of early OS contraction (∼−4 µm s^−1^ in Fig. 3) was greater than the magnitude of early SCS expansion (∼−3 µm s^−1^ in Fig. 3), and this was true in all subjects at all eccentricities. These differences suggest that, although the OS and SCS responses have opposite signs, they are not fully complementary to each other.

Measurable functional responses of the OS and SCS were observed for all subjects at all eccentricities 2° to 10° (see Fig. 4). The absolute maximum velocity of OS and SCS varied among subjects, but tended to fall with increasing eccentricity.

Responses were summarized by identifying extrema between 0 ms and 20 ms and computing the mean velocity between 20 ms and 40 ms. Identification of extrema (maximum or minimum velocity) was motivated by the observation that early responses had a visible trough (OS and CZ) or peak (SCS). Resulting summaries are shown in Figure 5, with parameters determined at the same eccentricity offset horizontally for visibility. Error bars indicate the standard deviation among subjects. Of the six parameters computed, three exhibited significant correlation with eccentricity: OS early minimum (*P* = 0.018), SCS early maximum (*P* = 0.032), and SCS late mean (*P* = 0.034).

To assess the reciprocity between changes in the OS and SCS, we first integrated the relative length changes of the OS and SCS and then added them together. If the two changes were reciprocal, then the sum of the two would remain near zero. Instead, we found that the sum, which corresponds with the ISOS to RPE distance, decreased over the first ∼20 ms and then increased thereafter. The pattern of changes resembled that of the OS itself, but with lower magnitude. To further reduce noise and verify that this effect was real, we averaged together the integrated and summed responses from 2° and 4° in each subject. The resulting responses from all four subjects are plotted in Figure 6, and clearly show a lack of reciprocity and overall contraction and then expansion of the distance between ISOS and RPE.

## Discussion

In these results, six distinct aspects of the response were apparent. Hypothetical attribution of the early OS contraction and later OS elongation to separate mechanisms^5^ suggests that the early (≤20 ms) and late (≥20 ms) stages of all the responses should be treated separately. In previous work,^8^^,^^13^ we quantified these stages using the maximum absolute velocity of the early stage and the mean velocity between 20 ms and 40 ms, and we follow the same approach here. The ranges described in Table are due to variation among subjects and eccentricity, and the averages are across both. These six aspects are illustrated in Figure 5.

**Table.** Summary of ORG Responses

	Early (µm/s)				Late (µm/s)		
	OS	SCS	CZ		OS	SCS	CZ
Mean	−3.1	2.0	−1.4		1.4	−0.8	0.6
Min	−1.6	0.5	−0.4		0.7	−0.4	0.0
Max	−5.2	3.0	−2.3		2.0	−1.2	1.3

Early (<20 ms) and late (20–40 ms) stimulus-evoked velocities from each of the outer retinal compartments. For each of these ORG parameters, values were averaged over eccentricities. The *mean* values are further averaged across subjects, while the *min* and *max* indicate the lowest and highest absolute velocities (speeds) across subjects. Nonzero values for the CZ show that OS and SCS velocities are not reciprocal. Because the semi automated segmentation was performed twice, once for the OS and once for the SCS. The sums of mean OS and SCS responses may differ from mean CZ responses depite their logical equivalence.

The observations of early OS contraction and late OS elongation (Table) corroborate similar findings from numerous groups.^2^^,^^4^^,^^5^^,^^8^ The remaining results in Table include three key findings: (1) the space between COST and RPE (SCS) exhibits a light-evoked change in thickness consisting of a rapid ∼20 ms of expansion followed by a slower contraction; (2) the space between the ISOS and RPE (CZ) exhibits changes more like the OS itself, consisting of a rapid contraction followed by elongation; and (3) both components of the latter are smaller in magnitude than those of the OS change. All of these are visualized in Figure 6. To our knowledge, this is the first report of rapid light-evoked changes in SCS and CZ directly measured in the living human eye, though the results do not conflict with previous findings by other groups in animal models or slower time scales. Changes in the optical path length of the CZ and SCS may involve the movement of water around the ECM or between the ECM and the cells that enclose it. Thus, it will be useful to review some of what is known about water movement in the outer retina. Importantly, the dynamics reported here occur within the first 50 ms following stimulus onset and therefore may represent rapid structural responses rather than slower transepithelial fluid transport mechanisms described in minute-scale studies.

### Water Movement in the SRS and Light-Evoked Hydration of ECM

The response reported in the present study occur on millisecond timescales (≤50 ms) and therefore may reflect rapid structural or osmotic processes intrinsic to the photoreceptor and adjacent ECM. Light-evoked hydration of the SRS has been reported in frogs^20^ and chicks,^30^^,^^22^ but over substantially longer time scales (seconds to minutes), so direct equivalence of the phenomena cannot be assumed. These comparisons are therefore intended as conceptual context rather than mechanistic proof of transepithelial fluid transport during the time window measured here. Previous physiological and diffusion-based imaging studies have also reported light-dependent changes in the SRS in animal models, suggesting dynamic outer retinal fluid redistribution.^33^^,^^34^ If we assume our subjects’ SRS hydrate by a fraction similar to the chick, 2.5%,^22^ and an ISOS-RPE distance of 40 µm, we would expect to see elongation of the space of ∼400 nms, which is within the range of CZ elongation we observed, 0 µm s^−1^ to 1.3 µm s^−1^ (Table).

### Synthesis of Results With Prior Work

In the past 15 years, many investigators have used OCT to study light-evoked changes in the outer retina. Most of these efforts have focused on changes in the OS specifically, but a few have noted changes outside of the OS. We have previously reported rapid changes in the amplitude of the OCT signal after light stimuli that are consistent with contraction of SCS around 0.1 second in cones^3^ and 1.0 second in rods.^6^ These consisted of apparent movement of an aspect of the RPE band inward, toward the ISOS, after stimuli. Recently Tan et al. (2024) reported light-evoked changes in the rat SRS^24^ on time scales of hundreds of milliseconds, using OCT phase. They defined the SRS thickness as the distance between the ELM and the RPE. In the OCT image of the rod-dominant rat retina, contributions from the ROST and RPE cannot be visually distinguished. However, an unsupervised learning method was used to differentiate these based on light-evoked phase changes. Some of the pixels in the ROST–RPE complex were shown to exhibit movement, relative to the inner segment/OS, similar to what has been shown in human rods and cones using AO-OCT: an OS elongation occurring over hundreds of milliseconds followed by a contraction lasting seconds and another elongation lasting tens of seconds. Other pixels, thought to originate from the RPE, exhibited slower, monotonic movement away from IS/OS over tens of seconds. The investigators attributed the latter to slow, light-evoked expansion of the SRS.

Other investigators have measured light-evoked changes outside of the photoreceptors over longer time scales. Abramoff et al. (2013) used the term OS equivalent length to describe what we here have called the CZ.^35^ They showed that the CZ contracts between 6 minutes and 20 minutes and expands between 20 minutes and 30 minutes after light onset. Li et al. (2016) showed differences between dark- and light-adapted mouse retina.^36^ They reported an overall light-dependent swelling of the outer retina, bounded by ELM and the choroid, between 15 minutes and 120 minutes after light onset.

Lu et al. measured such effects in humans between 20 seconds and 30 minutes following a bright stimulus flash that bleached 96% of rhodopsin. Rod OS (ISOS–ROST distance) was found to elongate 400 nm to 800 nm over the first 6 minutes following the bleach, and return to baseline over the next 20 minutes. They reported parallel changes in the distance of the RPE from the ISOS during recovery from the bleach. This distance, which corresponds with our CZ, exhibited a triphasic response, elongating between 0.33 minutes and 3 minutes, contracting between 3 minutes and 15 minutes, and elongating again to baseline between 15 minutes and 30 minutes.

Three studies by Messner et al.^37^^–^^39^ used commercial OCT to measure slow, light-evoked movements of outer retinal layers. These studies showed a slow contraction of the CZ between 1 minute and 5 minutes after light onset, which could be consistent with a long recovery of baseline SRS volume after its light-induced hydration. They also reported a contraction of the SCS between 1 minute and 10 minutes after light onset.

### Potential Limitations

The present measurements were acquired using a research-grade OCT system optimized for high-speed phase stability and precise stimulus synchronization. While the velocity-based ORG framework is not inherently restricted to this system, several aspects of the current implementation depend on capabilities that are not standard in commercial clinical OCT devices. In particular, integration of a synchronized stimulus channel and phase-stable acquisition were essential for reliable multi layer ORG measurements. Translation to clinical platforms would therefore require hardware adaptation, such as incorporation of a stimulus delivery pathway, which could potentially be implemented through modification of the fixation channel.

Additionally, mechanical stabilization using a bite bar was used to minimize motion artifacts. Such stabilization may not be feasible in routine clinical settings, and the robustness of velocity-based ORG under conventional chin-rest stabilization or purely algorithmic motion correction requires further validation. However, preliminary unpublished results from our team suggest that chin-rest stabilization may be sufficient for velocity-based ORG measurements.

The velocity-based ORG signals analyzed in this study were derived from discretely sampled, phase-based measurements and are therefore subject to uncertainty arising from finite temporal sampling, phase noise, segmentation variability, and residual eye motion. As a result, precise single-point peak timing cannot be resolved beyond the effective temporal resolution and noise floor of the acquisition and processing pipeline. To mitigate these effects, response timing was evaluated using predefined temporal windows rather than discrete peak times. This window-based approach reduces sensitivity to sampling jitter and noise and ensures that reported timing metrics reflect reproducible response features rather than isolated extrema. Nevertheless, fine timing differences on the order of a few milliseconds should be interpreted cautiously. Moreover, the noise in single trials is clearly higher than the noise in the averages (compare Figs. 2 and 3). This highlights the need for averaging multiple trials at iso-eccentric locations. In future work, we will investigate sources of noise and the possibility of modifying the instrument (e.g., increasing the B-scan rate) to reduce them. While the reported ORG metrics are quantitative and reproducible, their interpretation is limited by biological variability, phase noise, and temporal resolution, and they should therefore be viewed as comparative rather than high-precision absolute measurements.

Multi-layer velocity-based ORG analysis depends critically on sufficient axial resolution to isolate individual retinal bands. If adjacent reflectors fall within the same axial point spread function, the measured complex OCT signal represents a superposition of contributions from multiple layers, and phase-derived velocity estimates may reflect the angle of weighted phasor sums rather than motion of a single anatomical interface. Differences in axial resolution across OCT systems should therefore be considered when comparing multi-layer ORG performance between platforms.

## Conclusions

In this work, we have shown that visible stimuli cause relative movement among multiple layers in the outer retina. These results suggest that three compartments change in thickness after stimulation: the OS of the photoreceptor contracts briefly and then elongates; the supracone space, between the OS tip and the RPE, elongates briefly and then contracts; and the CZ, between the ISOS and RPE, contracts briefly and then elongates, both with lower amplitude than the OS.

The first of these observations confirms previous reports of OS deformation after light stimuli. The latter two observations have not been reported before, but are consistent with reported light-dependent hydration of the ECM in the SRS. Because those hydration changes take place in the context of water movement through the retina, and because water clearance from the RPE is known to be impacted by disease, the ability to measure them noninvasively may provide a noninvasive probe of outer retinal structural and osmotic dynamics, which may be altered in disease.
